# The F238L Point Mutation in the Cannabinoid Type 1 Receptor Enhances Basal Endocytosis via Lipid Rafts

**DOI:** 10.3389/fnmol.2018.00230

**Published:** 2018-07-05

**Authors:** Melanie Wickert, Keri L. Hildick, Gemma L. Baillie, Ruth Jelinek, Alejandro Aparisi Rey, Krisztina Monory, Miriam Schneider, Ruth A. Ross, Jeremy M. Henley, Beat Lutz

**Affiliations:** ^1^Institute of Physiological Chemistry, University Medical Center of the Johannes Gutenberg University, Mainz, Germany; ^2^School of Biochemistry, University of Bristol, Bristol, United Kingdom; ^3^Faculty of Medicine, University of Toronto, Toronto, ON, Canada; ^4^Institute of Psychopharmacology, Central Institute of Mental Health, Medical Faculty Mannheim, Heidelberg University, Mannheim, Germany; ^5^Department of Psychology, University of Heidelberg, Heidelberg, Germany; ^6^German Resilience Center (DRZ), University Medical Center of the Johannes Gutenberg University, Mainz, Germany

**Keywords:** CB1, endocytosis, lipid raft, axonal polarization, point mutation

## Abstract

Defining functional domains and amino acid residues in G protein coupled receptors (GPCRs) represent an important way to improve rational drug design for this major class of drug targets. The cannabinoid type 1 (CB1) receptor is one of the most abundant GPCRs in the central nervous system and is involved in many physiological and pathophysiological processes. Interestingly, cannabinoid type 1 receptor with a phenylalanine 238 to leucine mutation (CB1F238L) has been already linked to a number of both *in vitro* and *in vivo* alterations. While CB1F238L causes significantly reduced presynaptic neurotransmitter release at the cellular level, behaviorally this mutation induces increased risk taking, social play behavior and reward sensitivity in rats. However, the molecular mechanisms underlying these changes are not fully understood. In this study, we tested whether the F238L mutation affects trafficking and axonal/presynaptic polarization of the CB1 receptor *in vitro*. Steady state or ligand modulated surface expression and lipid raft association was analyzed in human embryonic kidney 293 (HEK293) cells stably expressing either wild-type cannabinoid type 1 receptor (CB1wt) or CB1F238L receptor. Axonal/presynaptic polarization of the CB1F238L receptor was assessed in transfected primary hippocampal neurons. We show that *in vitro* the CB1F238L receptor displays increased association with lipid rafts, which coincides with increased lipid raft mediated constitutive endocytosis, leading to a reduction in steady state surface expression of the CB1F238L receptor. Furthermore, the CB1F238L receptor showed increased axonal polarization in primary hippocampal neurons. These data demonstrate that endocytosis of the CB1 receptor is an important mediator of axonal/presynaptic polarization and that phenylalanine 238 plays a key role in CB1 receptor trafficking and axonal polarization.

## Introduction

The cannabinoid type 1 (CB1) receptor is an attractive target for therapeutic drugs because it plays an important role in the regulation of numerous physiological processes, including neural development (Maccarrone et al., 2014), the regulation of synaptic processes (Soltesz et al., 2015) and of various behaviors, e.g., learning and memory (Lutz, 2009; Ruehle et al., 2012), food intake (Silvestri and Di Marzo, 2013), and addiction (Moreira et al., 2015). It is also associated with many psychiatric disorders and neuropathological conditions (Pacher et al., 2006). In all these processes, CB1 receptor is key in the retrograde negative feedback of neurotransmission that the endocannabinoid system exerts. Additionally, the ample expression of this receptor in many brain areas and neural cell types makes the CB1 receptor a promising candidate in the treatment of pathological/dysregulated neuronal processes. An important approach for rational drug design is to identify residues and domains that are crucial for receptor function. Within the CB1 receptor there are defined domains involved in ligand binding (McAllister et al., 2003; Kapur et al., 2008; Shim et al., 2011), switching between active and inactive form (Nie and Lewis, 2001; Ahn et al., 2013; Marcu et al., 2013; Scott et al., 2013), and G-protein binding (Shim et al., 2013). Specific phosphorylation sites of the C-terminal tail of the CB1 receptor have been implicated in the regulation of CB1 receptor endocytosis and post-endocytic trafficking (Garcia et al., 1998; Hsieh et al., 1999; Jin et al., 1999; Daigle et al., 2008). Furthermore, mutations in the second extracellular loop and helix 8 (Ahn et al., 2009, 2010), as well as truncation of the N-terminus (Andersson et al., 2003) have been shown to affect CB1 receptor forward trafficking.

Endocytosis is a fundamental process in the regulation of G protein coupled receptors (GPCRs), being particularly needed for the appropriate termination of the molecular cascade that is triggered upon receptor activation. Similar to many other GPCRs, the CB1 receptor is subjected to agonist induced and/or constitutive endocytosis, and is subsequently degraded in lysosomes or recycled back to the plasma membrane (reviewed Rozenfeld, 2011). Intriguingly, constitutive (D’Antona et al., 2006; Leterrier et al., 2006; McDonald et al., 2007) and/or activity dependent (Simon et al., 2013) endocytosis has also been reported to play a major role in CB1 receptor axonal polarization. However, how these compartment-specific differences arise remains to be clarified. CB1 receptor internalization is mediated by both clathrin coated pit and caveolae/lipid raft mediated endocytosis (Keren and Sarne, 2003; Wu et al., 2008). Clathrin-mediated endocytosis of the CB1 receptor proceeds via a canonical G-protein-coupled receptor kinase mediated pathway, whose activation recruits β-arrestin 2 and other clathrin adapter proteins to direct receptor endocytosis (Hsieh et al., 1999; Jin et al., 1999; Ahn et al., 2013). However, the events leading to caveolae/lipid raft mediated endocytosis are less well understood. Caveolae/lipid rafts are a subset of membrane domains, consisting of a unique combination of lipids, including cholesterol and sphingolipids (reviewed Parton and Simons, 2007). The CB1 receptor is present in lipid rafts (Sarnataro et al., 2005; Asimaki et al., 2011) and co-localizes and interacts with the scaffolding protein caveolin-1 (Bari et al., 2008). The CB1 receptor contains three cholesterol recognition domains, which may be important for association with lipid rafts (Epand et al., 2006). Indeed, mutation of a lysine (L402), present in one cholesterol recognition domain of the CB1 receptor into the corresponding residue (glycine) in the cannabinoid type 2 receptor (which is not associated with lipid rafts), reduces lipid raft association (Oddi et al., 2011). However, whether this mutation affects caveolae/lipid raft mediated endocytosis of the receptor has not been reported.

Recently, a point mutation induced in Fischer rat has been described by Schneider et al. (2015), in which the phenylalanine 238 in the transmembrane helix 4 (TMH4) of the CB1 receptor is substituted by a leucine (cannabinoid type 1 receptor with a phenylalanine 238 to leucine mutation, CB1F238L). Computational analyses suggested that the mutant receptor is more labile to undergo changes in transmembrane regions that are associated with receptor activation and can thus be described as hypersensitive. Consistent with this, Schneider et al. (2015) reported an increase in cannabinoid stimulated [^35^S]GTPγS binding of the mutant receptor. They also demonstrated reduced presynaptic transmitter release probability in electrophysiological experiments and increased risk taking, social play behavior and reward sensitivity in the mutant rats. Importantly, in this mutant, no significant changes in the endocannabinoid system were detected other than the mutation in the CB1 receptor (Schneider et al., 2015).

Here, we examined trafficking of the CB1F238L mutant receptor using human embryonic kidney 293 (HEK293) cells stably expressing hemagglutinin (HA)-tagged wild-type CB1 (HA-CB1wt) or HA-tagged CB1F238L (HA-CB1F238L) and transiently transfected primary hippocampal neurons. We demonstrate that the mutant receptor shows increased basal internalization in HEK293 cells, which depends on caveolae/lipid raft mediated endocytosis. This finding is consistent with an increased lipid raft association of the CB1F238L receptor. Finally, the mutant receptor shows an increased surface polarization towards the axon in primary hippocampal neurons.

## Materials and Methods

### pcDNA3-HA-CB1 Constructs

BamHI and NotI restriction sites, a Kozak sequence as well as an N-terminal human influenza HA tag were added to the rat CB1wild-type (CB1wt) or rat CB1F238L sequence by PCR using the following primers: forward GCGGATCCACCATGGCATACCC-ATATGATGTCCCCGACTACGCGAAGTCGATCCTA-GATGGCCTTG, reverse GGCGC-GGCCGCTCACAGAGCCTCGGCGGA. The resulting sequence was cloned into the expression vector pcDNA3 using the restriction sites BamHI and NotI.

### Cell Lines Stably Expressing HA-CB1

HEK293 cells were grown in DMEM (Invitrogen) containing 10% fetal calf serum (A15-101, PAA The Cell Culture Company), Penicilin/Streptamycin, sodium butyrate and non-essential amino acids (all from Gibco Life Technologies). To establish cell lines stably expressing HA-CB1wt or HA-CB1F238L HEK293 cells were transfected with 24 μg of linearized DNA using Lipofectamine 2000 (Invitrogen) following the manufacturer’s instructions. Forty-eight hours after transfection, selection medium containing 1 mg/ml G418 (Geneticin) was added to the cells. To generate clones of resistant cells a limited dilution approach in 96-well plates was used. Clones were picked from those wells that originally contained only a single cell. To examine homogenous expression of HA-CB1 in the picked colonies immunocytochemistry was performed using a monoclonal antibody against the HA epitope of the receptor. Expression levels of HA-CB1 were determined by western blot analysis using a monoclonal antibody against the HA epitope of the receptor and subsequent normalization against actin. Those clones were chosen which showed intermediate receptor expression as compared to all clones which were generated. Furthermore, the chosen clones showed similar expression levels for both lines.

### Primary Hippocampal Neurons

Embryonic rat hippocampal neurons were prepared as described (Girach et al., 2013). At 5 days *in vitro* (DIV) cells were transfected with 0.75 μg of HA-CB1wt or HA-CB1F238L constructs using Lipofectamine 2000 according to manufacturer’s instructions. After 48 h cells were used for the polarization assay. Because our work is not recovery halothane anesthesia and immediate euthanasia not involving pain, it is designated as schedule 1 by the Home Office and it does not need Home Office licensing. All our work is overseen by, and fully conforms to the University of Bristol and UK ethical guidelines.

### Immunocytochemistry

HEK293 cells stably expressing HA-CB1wt or HA-CB1F238L were seeded on poly-L-lysine (Sigma) coated coverslips. After 48 h cells were washed in PBS and fixed in 4% PFA in PBS for 10 min. After washing with PBS cells were permeabilized with 0.05% Triton-X-100 in PBS for 5 min. After blocking with 4% goat serum in PBS for 15 min primary antibody (HA.11 16B12, MMs-101P, Covance 1:1000) in 4% goat serum was added for 2 h. After two 5 min washes with PBS, Alexa-488 conjugated secondary antibody (A-11008 Life Technologies, 1:1000) in 4% goat serum was added for 30 min. Subsequently cells were washed once for 5 min and nuclei were stained with DAPI (Hoechst) for 5 min. Cells were washed once in PBS and mounted on Mowiol. All steps were carried out at room temperature.

### Trypsin Protection Assay

Trypsin protection assay was performed as described (Grimsey et al., 2010) with slight modifications. Stable HEK293 cells were placed in serum free medium (SFM) 1 h prior to the experiment. For endocytosis experiments, cells were treated either with 100 μM WIN-55212-2 (BN0544, BioTrend Chemicals AG) or 100 μM SR141716 (generously provided by the NIMH Chemical Synthesis and Drug Supply Program) in SFM for 45 min, with 20 μM PitStop2™ (Abcam) for 15 min or 5 mM methyl-β-cyclo-dextrin (MβCD; Sigma) in SFM for 30 min. WIN-55212-2, SR141716 and PitStop2™were dissolved in DMSO, MβCD was dissolved in SFM. All treatments were matched with appropriate vehicle conditions. After treatment the medium was removed and either 2 ml of versene (15040-033 Gibco Life Technologies) as control or trypsin solution (25300-054 Gibco Life Technologies) were added. After 4 min 10 ml of cold DMEM containing 10% fetal calf serum, Penicillin/Streptomycin, sodium butyrate and non-essential amino acids were added to stop the enzymatic reaction. Cells were pelleted and washed two times with cold PBS to remove any residual trypsin. The pellet was resuspended in lysis buffer (150 mM NaCl, 1 mM EDTA, 1 mM EGTA, 20 mM Tris, pH 7.5, 1% CHAPS, HaltTM Protease and Phosphatase Inhibitor Cocktail, Thermo Scientific) and lysed by rotating over night at 4°C. Subsequently nuclei and cell debris were removed by centrifugation at 1000 *g* and 4°C for 10 min. Protein concentration of the supernatant was determined using a Bradford protein assay (Biorad). The lysate was analyzed by western blot. Signals for HA were normalized against tubulin or actin and percent intracellular CB1 receptor was calculated by dividing normalized HA signal in trypsin treated cells by normalized HA signal in versene treated cells and multiplied by a hundred. In conclusion percent surface CB1 receptor was calculated by subtracting percent intracellular CB1 receptor from a hundred.

### Lipid Raft Preparation

To prepare lipid rafts from stable HEK293 cells, a detergent free protocol using a discontinuous sucrose gradient was used. Cells were placed in SFM 1 h prior to the experiment. Medium was removed and cells were scraped into cold PBS and pelleted at 118 g and 4°C for 5 min. The supernatant was removed and the cell pellet was resuspended in 900 μl ice cold 45% sucrose in TBS. After sonification with three strokes for 10 s each (Bandelin Sonopuls HD60), the homogenate was passed through a 25G cannula for 20 times. Eight-hundred microliter of the homogenate was placed in an ultracentrifuge tube and overlaid with 2700 μl of 30% sucrose in TBS and 500 μl of 5% sucrose in TBS. The sucrose gradient was centrifuged in a Beckmann ultracentrifuge using a SW40TI rotor at 4°C and 100,000 *g* for 24 h to 26 h. Subsequently eight 400 μl fractions and one 800 μl fraction were collected from the top to the bottom. The 800 μl bottom fraction included the resuspended pellet. 8 μl of each fraction were analyzed by western blot. HA signals were normalized against caveolin-1 for each fraction and normalized HA values from fractions 2–4 were chosen as lipid raft fractions. All other fractions were considered as non-raft fractions.

### Western Blot

If not stated otherwise 20 μg of protein were separated by electrophoresis on a 10% polyacrylamide/SDS gel. Subsequently proteins were blotted to a nitrocellulose membrane using a Biorad wet blot system. Membranes were stained with Ponceau for 5 min to control for successful transfer of proteins to the membrane. Subsequently the membranes were blocked with 5% non-fat dry milk in TBST for 1 h at room temperature and primary antibody (HA.11 16B12, MMs-101P, Covance 1:1000; T9026 mouse anti tubulin, Sigma Aldrich, 1:200,000; 04–1040 rabbit anti actin, Millipore, 1:1000; SC-894 rabbit anti caveolin-1, Santa Cruz, 1:1000) diluted in 5% non-fat dry milk in TBST incubated over night at 4°C. Primary antibody was removed and after three 10-min washes with TBST, horseradish peroxidase coupled secondary antibody was added in 5% non-fat dry milk and incubated for 30 min at room temperature. After two 10-min washes with TBST and one 10-min wash with PBS membranes were incubated with ECL solution (Amersham ECLTM Prime Western Blotting Detection Reagent) according to the manufacturer’s instructions and imaged with the Peqlab Fusion-SL system using the fusion software version 15.16 by Vilber Lourmat. The molecular weight of western blot bands was calculated with the same imaging software. Quantification of western blot bands was performed using the bio1D software version 15.02 by Vilber Lourmat.

### Equilibrium Binding Assay

HEK293 cells stably expressing HA-CB1wt or HA-CB1F238L were scraped into cold PBS and pelleted with 2000 *g* at 4°C for 4 min. After cells were resuspended in cell buffer (50 mM Tris HCl, 1 mM EDTA and 3 mM MgCl_2_), the protein content was measured by Bradford assay (Biorad). Equilibrium binding assays were carried out using the cannabinoid receptor agonist [^3^H]CP-55940, at a concentration of 0.7 nM. 1 mg/ml BSA and 50 mM Tris buffer was used in a total assay volume of 500 μl containing 0.01% DMSO. Binding was initiated by adding 1 mg/ml cells. Assays were incubated at 37°C for 1 h. The reaction was stopped by the addition of ice cold wash buffer that contained 50 mM Tris buffer and 1 mg/ml BSA and vacuum filtration using a 24-well sampling manifold Brandel cell harvester and Whatman GF/B glass-fiber filters that had been soaked in the same wash buffer at 4°C for at least 24 h. Each reaction tube was washed six times with a 500 μl aliquot of buffer. The filters were then oven dried for 1 h, and then placed in 5 ml scintillation vials with 4 ml of scintillation fluid. Radioactivity was quantified by liquid scintillation spectrometry. Specific binding is defined as the difference between the binding that occurred in the presence and absence of 1 μM CP-55940 and varied between 70%–90% of the total binding.

### Surface Polarization Assay

Surface polarization in primary hippocampal neurons was evaluated by quantitative immunofluorescence analysis by confocal microscopy in accordance with previous publications (see Sampo et al., 2003; Silverman et al., 2005; Leterrier et al., 2006; McDonald et al., 2007). Briefly, 20 μm long segments of dendrites and axons were manually traced using the polygon selection tool in ImageJ. Mean surface fluorescence intensity along each segment was then measured (in the corresponding channel), with an average of 3–5 dendritic and axonal segments each outline per neuron. Dendrites and axons were identified by microtubule-associated protein 2 (MAP2) positive or negative counterstaining. Morphological classification was facilitated by restricting analysis to DIV 7–9 neurons, as these younger neurons have established polarity but do not yet display complex dendritic arborization, making the long thin axonal projections easily identifiable. Segments proximal to soma are termed “proximal”; segments near the growth edge are termed “distal” and segments mid-way along the length of the dendrite are termed “intermediate.” For each condition, typically 10–15 neurons each from at least three separate experiments were used. Each data set was evaluated using the D-Agostino and Pearson omnibus normality test to confirm normal population distribution (suitable for parametric statistical analysis).

### Quantitative Immunofluorescence Analysis

Transfected neurons were placed in pre-warmed HBS buffer (25 mM HEPES, 137 mM NaCl, 5 mM KCl, 15 mM glucose, 1.5 mM CaCl_2_, 1.5 mM MgCl_2_, pH7.4, osmolarity adjusted to within ± 10 mOsM of the culture medium) and were allowed to adjust for 5 min at room temperature. To label surface CB1 receptor primary mouse monoclonal HA antibody (H3663 Sigma 1:200) in HBS buffer was added to the coverslips for 20 min. After washing the coverslip three times in HBS cells were fixed with pre warmed paraformaldehyde solution (4% paraformaldehyde, 5% sucrose in PBS). After 15 min the coverslips were washed three times in PBS and residual paraformaldehyde was quenched by adding 50 mM ammonium chloride in PBS for 5 min. Subsequently coverslips were washed three times with PBS and blocked with 10% horse serum in PBS for 10 min. Donkey anti mouse Cy3-conjugated secondary antibody (715-165-151 Jackson Immuno 1:600) was applied in 5% horse serum in PBS for 45 min. After three washes with PBS cells were permeabilized with 0.1% TritonX-100 in PBS for 10 min, washed two times with PBS and blocked with 10% horse serum for 20 min. Primary mouse monoclonal antibody against HA (1:400) and rabbit polyclonal antibody against MAP2 (M3696 Sigma 1:600) in 5% horse serum were added to the coverslips for 1 h. After washing three times with PBS, donkey anti-mouse Cy2 coupled (715-225-150 Jackson Immuno 1:600) and donkey anti-rabbit Cy5 coupled (715–225–152 Jackson Immuno 1:600) secondary antibodies were added for 1.5 h. Subsequently coverslips were washed three times in PBS and mounted in Fluoromount (Sigma). All steps were performed at room temperature.

### Confocal Microscopy

Neurons were imaged using the 40× oil objective of a Zeiss LSM510 UV META Axiovert 200M laser scanning microscope and the Zeiss LSM Data Server software. Cy2 was excited with an Argon/2 laser at 488 nm and emission was detected using a 505–530 nm band pass filter (displayed as green channel). Cy3 was excited with a He/Ne laser at 543 nm and emission was detected using a 560–615 nm band pass filter (displayed as red channel). Cy5 was excited with a He/Ne laser at 633 nm and emission was detected using a 650 nm long pass filter (displayed as blue channel). Z-stacks of four optical slices per cell were recorded with 4× averaging, 1024 × 1024 pixels and 8-bit depth. If necessary multiple pictures per cell were collected to capture the entire neuron.

### Analysis

Quantification of fluorescence was performed using ImageJ software. Maximum projections were used to quantify surface CB1 receptor fluorescence. 20 μm × 1 μm regions of interest (ROIs) from dendrites (MAP2+) and proximal, intermediate and distal parts of the axon (MAP2−) were selected and fluorescence intensity was measured for the surface CB1 receptor. To avoid selection bias, ROIs were chosen in merged images showing only the total CB1 receptor and MAP2 channel data. Average axonal surface CB1 receptor was calculated by taking the average of surface CB1 receptor in proximal, intermediate and distal parts of the axon. Average dendritic surface CB1 receptor was calculated by taking the average of surface CB1 receptor in two to three different dendrites of one cell. The surface CB1 receptor polarization index (A/D ratio) for each cell was determined by dividing average axonal surface CB1 receptor by average dendritic surface CB1 receptor. To measure the gradient of surface CB1 receptor expression from the proximal to the distal axon values for the intermediate and distal axon were expressed as percent of the value for the proximal axon. The experimenter was blind to the genotype throughout the imaging and analysis procedure to avoid any bias.

### Statistics

Two-way ANOVA or repeated measures ANOVA and Bonferroni’s *post hoc* test, student’s *t*-test and one sample *t*-test were performed using GraphPad Prism version 4.00 for Windows (GraphPad Software, San Diego, CA, USA). Data are expressed as mean ± SEM.

## Results

### Characterization of HEK293 Cells Stably Expressing HA-CB1wt or HA-CB1F238L

**Figure 1:**
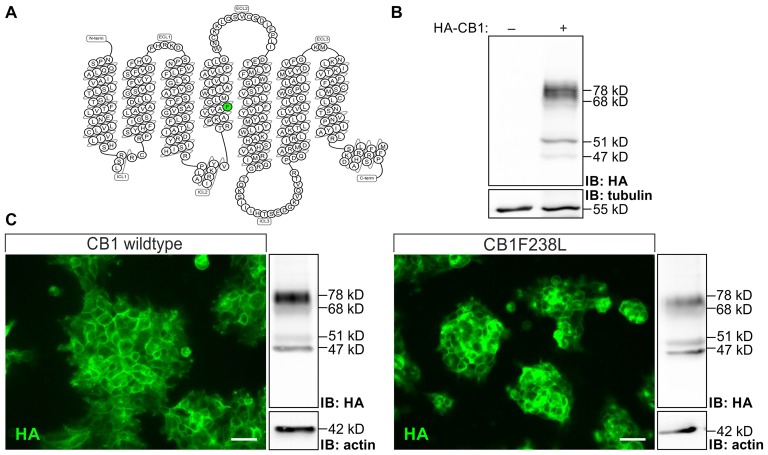
Characterization of human embryonic kidney 293 (HEK293) cells stably expressing hemagglutinin-tagged cannabinoid type 1 wild-type (HA-CB1wt) or HA-cannabinoid type 1 receptor with a phenylalanine 238 to leucine mutation (CB1F238L). **(A)** Snake plot of the rat wild-type CB1 receptor with phenylalanine 238 labeled in green. Source: gpcrdb.org/ (Pándy-Szekeres et al., 2018). **(B)** Relative expression levels of HA-tag signal in transfected and untransfected HEK293 cells. HA-tag signal in HEK293 cells not transfected or stably transfected with pcDNA3-HA-CB1wt were detected by western blot analysis. Four bands were detected at 78 kD, 68 kD, 51 kD and 47 kD in transfected cells. All four bands detected in the western blot are specific as they do not appear in untransfected HEK293 cells. **(C)** Subcellular localization was examined by immunostaining using a monoclonal anti-HA antibody. Expression levels were quantified by western blot analysis using a monoclonal anti-HA antibody and normalizing to actin. Two clones with moderate and similar HA-CB1 expression levels where chosen. IB, Immuno blot. (Scale bar: 20 μm).

HEK293 cells stably expressing HA-CB1wt or HA-CB1F238L were quantified by western blot analysis and clones with intermediate and similar expression levels between genotypes were selected. Four bands of 78 kD, 68 kD, 51 kD, 47 kD were observed in the western blot for HA-CB1 which were shown to be specific as compared to untransfected cells (Figure 1). The 51 kD band represents the putative CB1 monomer. Additional bands visible in the same lane may represent receptor dimers (Wager-Miller et al., 2002), glycosylated/deglycosylated (De Jesús et al., 2006) or other post-translationally modified receptors.

**Figure 2:**
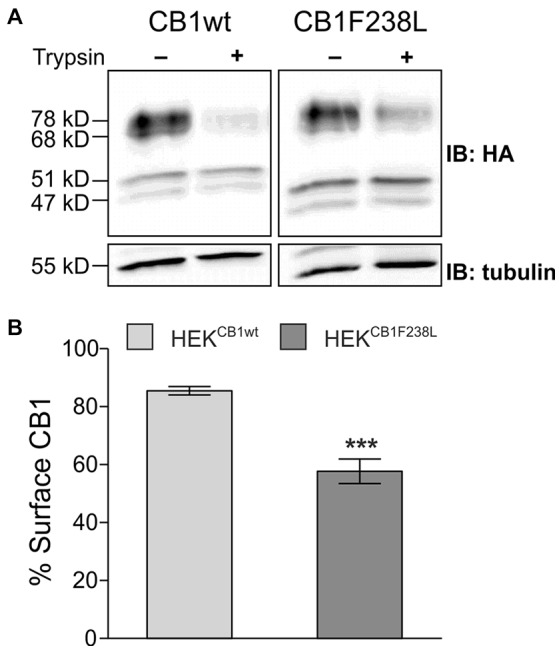
Surface expression of HA-CB1F238L compared with HA-CB1wt. **(A)** HEK293 cells stably expressing HA-CB1wt or HA-CB1F238L were treated with trypsin or versene (control). Trypsin is able to cleave the extracellular HA-tag. Thus, control samples show total CB1 receptor amount, whereas trypsin treated samples show intracellular CB1 receptor amount. **(B)** Surface HA-CB1 was calculated and expressed as percent of total HA-CB1. We found 85.49% (±1.44) of HA-CB1wt, but only 57.68% (±4.26) of HA-CB1F238L to be located to the plasma membrane. (Student’s *t*-test. Data are presented as the mean ± SEM of *n* = 7 independent experiments. ***p < 0.001).

Using a trypsin protection assay and western blot analysis, we quantified the surface expression of the wild-type and mutant CB1 receptors. In short, trypsin treatment of cells leads to the cleavage of the HA-tag of surface CB1 receptor, whereas intracellular receptor remains unaffected. Thus, surface and intracellular receptor can be distinguished by HA immunoreactivity. Trypsin treatment reduced the intensity of the two higher molecular bands but did not affect the 47 kD and 51 kD bands (Figure 2A). This is in accordance with the observation by Grimsey et al. (2010). The authors state that the lower molecular bands are deglycosylated forms of the CB1 receptor located in the intracellular biosynthetic or degradation pathway, which protects them from trypsin cleavage (Grimsey et al., 2010). Additionally to the 68 kD band, which corresponds to the 64 kD band observed by Grimsey et al. (2010), we also observed 78 kD band, which might be a CB1 receptor species with different posttranslational modification. Both, the 68 kD and 78 kD bands were used to quantify surface CB1 receptor (Figure 2A). We found a significant difference in plasma membrane localization with 85 ± 1% of HA-CB1wt but only 58 ± 4% of HA-CB1F238L being expressed on the cell surface (Figure 2B).

### HA-CB1F238L Surface Expression Can Be Rescued by Inverse Agonist Treatment

**Figure 3:**
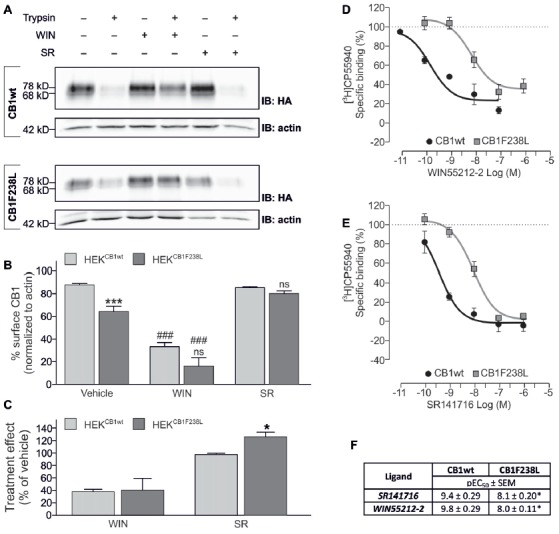
Effect of WIN-55212-2 and SR141716 on surface expression of HA-CB1F238L compared to HA-CB1wt. **(A)** HEK293 cells stably expressing HA-CB1wt or HA-CB1F238L were treated either with vehicle, 100 nM WIN-55212-2 or 100 nM SR141716 for 45 min. Surface expression was analyzed by a trypsin protection assay. **(B)** Strong internalization after WIN-55212-2 treatment was observed for both receptors. SR141716 treatment rescued surface expression of CB1F238L receptor back to wild-type levels. Surface HA-CB1 was calculated and expressed as percent of total HA-CB1 as described in “Materials and Methods” section. (Two way ANOVA and Bonferroni’s *post hoc* test. Data are presented as the mean ± SEM of *n* = 4 independent experiments. ***p < 0.001; n.s., *p* > 0.05 vs. wild-type; ^###^*p* < 0.001 vs. vehicle). **(C)** The effect of SR141716 on surface expression is significantly increased for CB1F238L receptor (Student’s *t*-test between genotypes. Data are presented as the mean ± SEM of *n* = 4 independent experiments. *p < 0.05). Competition binding experiments with radiolabeled CB1 receptor agonist [^3^H]CP55940 showed that the F238L mutation causes a decrease in the affinity of CB1 receptor **(D)** for the agonist WIN55212-2 and **(E)** for the inverse agonist SR141716. **(F)** In competition binding experiments with [^3^H]CP-55940 the CB1F238L receptor showed significantly reduced affinity for WIN-55212-2 as well as for SR141716. (Student’s *t*-test of pEC_50_. Data are presented as the mean ± SEM of *n* = 3–8 independent experiments. *p < 0.05).

To test if the altered surface expression of the F238L mutant is due to differences in internalization, we examined the endocytosis behavior of the CB1F238L receptor in response to agonist stimulation. The cannabinoid receptor agonist WIN-55212-2 is known to induce strong endocytosis of the CB1 receptor (Hsieh et al., 1999; Figure 3A). WIN-55212-2 treatment (100 nM, 45 min) induced strong internalization of both HA-CB1wt and HA-CB1F238L (Figure 3B) with no significant difference between wild-type and mutant receptors (Figure 3C). Interestingly, however, equilibrium binding assays showed a decreased affinity of the mutant receptor for WIN-55212-2 (Figures 3D,F). These data imply that the CB1F238L mutation might alter the binding affinity for WIN-55212-2. Conversely, inverse agonist treatment stabilizes the CB1 receptor at the surface (Grimsey et al., 2010). We therefore tested the effect of inverse agonist SR141716 on HA-CB1F238L surface expression (Figure 3A). One-hundred nanomolar SR141716 for 45 min rescued surface expression of the mutant receptor to wild-type levels (Figure 3B). As with WIN-55212-2, equilibrium binding assays showed a decreased affinity of the CB1F238L receptor for SR141716. This demonstrates that the higher effect that SR141716 has on the surface expression of the CB1F238L receptor (Figure 3F) is not due to an increased affinity of the mutant receptor for SR141716 (Figure 3E).

### Increased Basal Endocytosis of HA-CB1F238L Is Mediated by Caveolae/Lipid Rafts and Not by Clathrin Coated Pits

**Figure 4:**
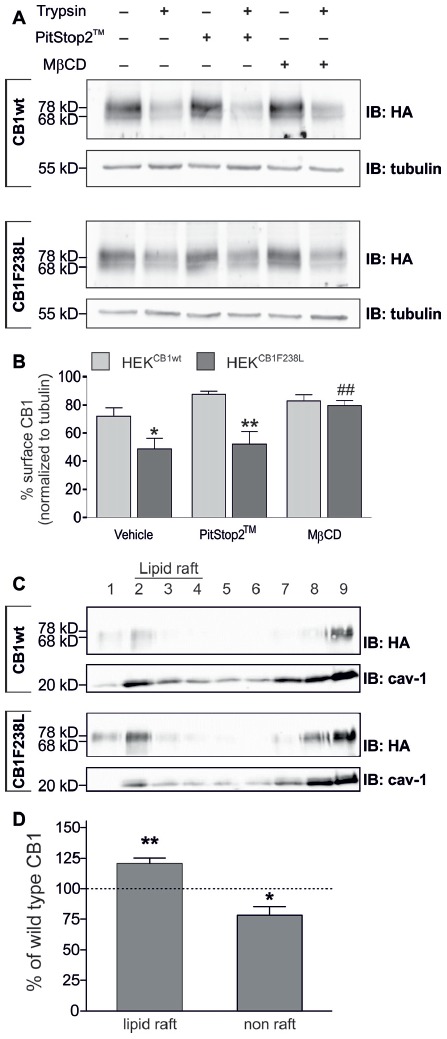
Surface expression of HA-CB1F238L compared with HA-CB1wt after inhibition of clathrin coated pit or caveolae mediated endocytosis. **(A)** HEK293 cells stably expressing HA-CB1wt or HA-CB1F238L were treated either for 15 min with 20 μM PitStop2™ to inhibit clathrin coated pit endocytosis or for 30 min with 5 mM methyl-β-cyclo-dextrin (MβCD) to inhibit caveolae mediated endocytosis. Surface expression was analyzed by a trypsin protection assay. **(B)** Only MβCD treatment significantly increased surface expression of CB1F238L receptor. (Two way ANOVA and Bonferroni’s *post hoc* test. Data are presented as the mean ± SEM of *n* = 4 independent experiments. Surface HA-CB1 was calculated and expressed as percent of total HA-CB1 as described in “Materials and Methods” section *p < 0.05; **p < 0.01; vs. wild-type; ^##^*p* < 0.01 vs. vehicle). **(C)** Lipid rafts were prepared from HEK293 cells stably expressing HA-CB1wt or HA-CB1F238L and analyzed by western blot. HA signals were normalized against caveolin and values for caveolin rich fractions 2–4 were chosen as lipid raft fractions, all others as non-raft fractions (see “Materials and Methods” section). cav-1, calveolin **(D)** Values for CB1F238L receptor were expressed as percentage of CB1wt receptor. (One sample *t*-test. Data are presented as the mean ± SEM of *n* = 7 independent experiments. *p < 0.05; **p < 0.01).

To test if basal endocytosis of the CB1 receptor mediates the decreased surface expression of HA-CB1F238L, we inhibited clathrin-mediated or caveolae/lipid raft-mediated internalization. Cells were treated either with 20 μM PitStop2™ for 15 min to inhibit clathrin-mediated endocytosis (Dutta et al., 2012), or with 5 mM MβCD for 30 min, a cholesterol depleting agent that disrupts caveolae/lipid rafts (Figure 4A). PitStop2™ treatment did not affect surface expression of the CB1F238L receptor. In contrast, MβCD treatment led to an increase in surface expression of the CB1F238L receptor consistent with decreased caveolae/lipid raft-mediated endocytosis of the mutant receptor (Figure 4B).

We next tested if CB1F238L is enriched in biochemical lipid raft preparations using a detergent free protocol in a discontinuous sucrose gradient (Figure 4C). We found a significant increase of CB1F238L receptor compared to the CB1wt receptor in the lipid raft fractions (Figure 4D).

### CB1F238L Receptor Shows Decreased Surface Expression in Dendrites and Increased Axonal Polarization

**Figure 5:**
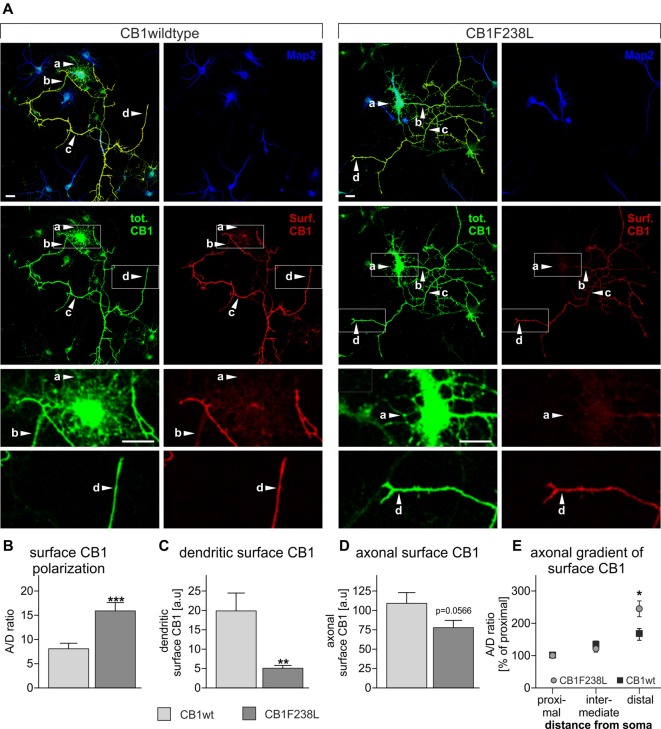
Axonal polarization of HA-CB1wt and HA-CB1F238L in primary hippocampal neurons. Immunocytochemistry was performed using primary rat hippocampal neurons which were transfected with either HA-CB1wt or HA-CB1F238L. **(A)** Surface HA-CB1 receptor is shown in red, total HA-CB1 receptor in green and microtubule-associated protein 2 (MAP2) in blue. Surface HA-CB1 receptor was quantified in 20 × 1 μm regions of interest (ROIs) in the dendrites (MAP2+; a), proximal axon (MAP2−; b), intermediate axon (MAP2−; c) and distal axon (MAP2−; d). **(B)** The polarization index (A/D) for surface CB1 receptor was determined by dividing average axonal surface fluorescence by average dendritic surface fluorescence (Scale bar: 20 μm). **(C)** There is a significant reduction in dendritic surface CB1F238L receptor. **(D)** In the axon, there is a small reduction in surface CB2F238L, which did not reach statistical significance though. (Student’s *t*-test. Data are presented as the mean ± SEM of 21 HA-CB1wt cells and 30 HA-CB1F238L cells of two independent experiments. **p < 0.01; ***p < 0.001). **(E)** Intermediate and distal axonal surface CB1 receptor is expressed as percent of surface CB1 receptor in the proximal axon (repeated measures ANOVA and Bonferroni’s *post hoc* test. Data are presented as the mean ± SEM of 19 HA-CB1wt cells and 26 HA-CB1F238L cells of two independent experiments. *p < 0.05).

Several studies have shown that selective removal of the CB1 receptor from the somatodendritic compartment, via a constitutive endocytic pathway is required to maintain proper receptor distribution and axonal surface polarization (Leterrier et al., 2006; McDonald et al., 2007; Simon et al., 2013). As the CB1F238L receptor shows increased constitutive endocytosis in HEK293 cells we next evaluated the surface distribution of CB1F238L in primary hippocampal neurons by quantitative immunofluorescence (Figure 5). Using the axonal polarization index (ratio of axonal to dendritic surface fluorescence; A/D ratio) we observed an almost two-fold difference on the polarity of the wild-type and mutant receptor (Figure 5B). We attribute this predominantly to a significant reduction of dendritic surface expression of CB1F238L (Figure 5C). Surprisingly, there was a non-significant trend to reduced axonal surface expression of CB1F238L (Figure 5D) but a significantly increased axonal gradient for the CB1F238L receptor towards the distal end of the axon as compared with the wild-type receptor (Figure 5E).

## Discussion

The F238L point mutation in the rat CB1 receptor has been reported to result in a hyperactive receptor on the biochemical as well as electrophysiological and behavioral level (Schneider et al., 2015). At the cell biological level we observed a reduction in surface expression of mutant receptor could be eliminated by treatment of the cells with the CB1 receptor inverse agonist SR141716. On the other hand, when cells were treated with the CB1 receptor agonist WIN-55212-2 there was no difference in induced internalization between wild-type and mutant receptor. This indicates an effect of the mutation on basal but not agonist induced internalization of the receptor. However, we cannot exclude that there might be a difference in the kinetics of agonist induced endocytosis.

Interestingly, equilibrium binding experiments showed a reduced affinity of the mutant receptor for SR141716 as well as for WIN-55212-2. However, although the CB1F238L receptor showed reduced affinity for SR141716, the inverse CB1 receptor agonist was still able to rescue surface expression of the CB1F238L receptor. The observation that exchanging an aromatic residue at position 238 of the CB1 receptor by a non-aromatic residue entails decreased affinity for SR141716 as well as for WIN-55212-2 is an interesting finding in itself. In fact, an aromatic microdomain between TMH 3-4-5-6 has been suggested to be the binding site for SR141716 as well as for WIN-55212-2 but not for CP-55940 (McAllister et al., 2003), for which, accordingly, we did not see any difference in affinity either (data not shown).

To further support the concept that the mutant receptor undergoes increased basal endocytosis, we inhibited the two internalization pathways that have been described for the CB1 receptor with MβCD (inhibitor of clathrin-mediated endocytosis) and PitStop2™ (inhibitor of caveolae/lipid raft mediated endocytosis). Although MβCD treatment has been reported to also affect clathrin coated pit budding under certain conditions (Rodal et al., 1999; Subtil et al., 1999), the increase that this compound triggers in surface expression of CB1F238L combined with the lack of effect under PitStop2™ treatment indicates that the CB1F238L receptor undergoes increased caveolae/lipid raft mediated rather than clathrin coated pit mediated endocytosis. Caveolae and lipid rafts are membrane domains characterized by a unique composition of lipids, including cholesterol and sphingolipids (Parton and Simons, 2007). They are thought to compartmentalize scaffolding and signaling molecules as well as playing a role in endocytosis (Allen et al., 2007). The CB1 receptor as well as the endocannabinoids 2-arachidonoyl glycerol (2-AG) and anandamide (AEA) have been reported to be targeted into lipid rafts (Sarnataro et al., 2006; Dainese et al., 2007; Rimmerman et al., 2008; Asimaki et al., 2011), and lipid raft disruption by MβCD has been shown to affect CB1 receptor ligand as well as G-protein binding (Bari et al., 2005a,b). Furthermore, in immunoprecipitation and colocalization studies the CB1 receptor has been shown to interact with caveolin-1, a protein enriched in lipid rafts and caveolae (Bari et al., 2008). In agreement with the increased endocytosis of the mutant receptor via caveolae/lipid rafts, biochemical lipid raft preparations revealed a significantly higher amount of CB1F238L receptor in the lipid raft fractions. However, the mechanism by which mutation of phenylalanine 238 affects lipid raft allocation remains to be examined. Possibly, the activation state or ligand binding of the receptor has an influence on its lipid raft allocation, as it has been suggested for other receptors, including the CB1 receptor (Chini and Parenti, 2004; Sarnataro et al., 2005, 2006; Allen et al., 2007). Another possibility is differential protein-protein interactions of the CB1F238L receptor with lipid raft scaffolding proteins, such as caveolin-1. Although a caveolin-1 interacting domain containing aromatic residues has been suggested many years ago (Couet et al., 1997), phenylalanine 238 in the CB1 receptor is not part of such a domain. Finally, it could also be possible that a differential interaction with membrane lipids, such as cholesterol, plays a role here even though phenylalanine 238 is not part of a cholesterol recognition amino acid consensus (CRAC; cholesterol-consensus binding motif) domain.

Given that endocytosis of the CB1 receptor has been described to be one crucial step for the polarization of this receptor to the axon (Leterrier et al., 2006; McDonald et al., 2007; Simon et al., 2013), we finally tested whether the mutant receptor polarizes differentially within the neuronal surface. For the CB1F238L receptor we found a significantly increased axonal polarization (especially towards the distal axon) due to a strong decrease in dendritic surface expression. All these findings correspond well to predictions made by the model by Simon et al. for a hyperactive receptor (Simon et al., 2013).

In conclusion, our study provides evidence that phenylalanine 238 plays a role in the allocation of the CB1 receptor into lipid rafts and in the receptor’s trafficking properties. It also hints at a possible mechanistic explanation for CB1 receptor axonal polarization and for the first time implicates non-clathrin-mediated constitutive endocytosis in regulating CB1 surface distribution. Also, the CB1F238L receptor provides a valuable tool to further study the role of caveolae/lipid rafts in CB1 receptor trafficking.

## Author Contributions

MW, KH, GB, AAR and RJ performed the research. MW, KH, GB, RR, JH and BL designed the research study. MS contributed essential reagents. MW, KH, GB and AAR analyzed the data. MW, KM and BL wrote the article.

## Conflict of Interest Statement

The authors declare that the research was conducted in the absence of any commercial or financial relationships that could be construed as a potential conflict of interest.

## Abbreviations

A/D ratio: polarization index for surface CB1 receptor axonal polarization
CB1: cannabinoid type 1 receptor
CB1F238L: cannabinoid type 1 receptor with a phenylalanine 238 to leucine mutation
CB1wt: wild-type cannabinoid type 1 receptor
CP-55940: 2-((1R,2R,5R)-5-hydroxy-2-(3-hydroxypropyl) cyclohexyl)-5-(2-methyloctan-2-yl)phenol
HA: hemagglutinin
MβCD: methyl-β-cyclodextrine
SR141716: 5-(4-chlorophenyl)-1-(2,4-dichlorophenyl)-4-methyl-N-piperidin-1-ylpyrazole-3-carboxamide
TMH4: transmembrane helix 4
WIN-55212-2: (11R)-2-methyl-11-(morpholin-4-ylmethyl)-3-((naphthalen-1-yl)carbonyl)-9-oxa-1-azatricyclo(6.3.1.0) dodeca-2,4(12),5,7-tetraene.

## References

[B1] Ahn K. H. Bertalovitz A. C. Mierke D. F. Kendall D. A. (2009). Dual role of the second extracellular loop of the cannabinoid receptor 1: ligand binding and receptor localization. Mol. Pharmacol. 76, 833–842. 10.1124/mol.109.057356 19643997 PMC2769047

[B2] Ahn K. H. Nishiyama A. Mierke D. F. Kendall D. A. (2010). Hydrophobic residues in helix 8 of cannabinoid receptor 1 are critical for structural and functional properties. Biochem 49, 502–511. 10.1021/bi901619r 20025243 PMC2904477

[B3] Ahn K. H. Scott C. Abrol R. Goddard W. A. III. Kendall D. A. (2013). Computationally-predicted CB1 cannabinoid receptor mutants show distinct patterns of salt-bridges that correlate with their level of constitutive activity reflected in G protein coupling levels, thermal stability, and ligand binding. Proteins 8, 1304–1317. 10.1002/prot.24264 23408552 PMC4872635

[B4] Allen J. A. Halverson-Tamboli R. A. Rasenick M. M. (2007). Lipid raft microdomains and neurotransmitter signalling. Nat. Rev. Neurosci. 8, 128–140. 10.1038/nrn2059 17195035

[B5] Andersson H. D’Antona A. M. Kendall D. A. Von Heijne G. Chin C. (2003). Membrane assembly of the cannabinoid receptor 1: impact of a long N-terminal tail. Mol. Pharmacol. 64, 570–577. 10.1124/mol.64.3.570 12920192

[B6] Asimaki O. Leondaritis G. Lois G. Sakellaridis N. Mangoura D. (2011). Cannabinoid 1 receptor-dependent transactivation of fibroblast growth factor receptor 1 emanates from lipid rafts and amplifies extracellular signal-regulated kinase 1/2 activation in embryonic cortical neurons. J. Neurochem. 116, 866–873. 10.1111/j.1471-4159.2010.07030.x 21214560

[B7] Bari M. Battista N. Fezza F. Finazzi-Agrò A. Maccarrone M. (2005a). Lipid rafts control signaling of type-1 cannabinoid receptors in neuronal cells. Implications for anandamide-induced apoptosis. J. Biol. Chem. 280, 12212–12220. 10.1074/jbc.M411642200 15657045

[B9] Bari M. Paradisi A. Pasquariello N. Maccarrone M. (2005b). Cholesterol-dependent modulation of type 1 cannabinoid receptors in nerve cells. J. Neurosci. Res. 81, 275–283. 10.1002/jnr.20546 15920744

[B8] Bari M. Oddi S. De Simone C. Spagnolo P. Gasperi V. Battista N.. (2008). Type-1 cannabinoid receptors colocalize with caveolin-1 in neuronal cells. Neuropharmacology 54, 45–50. 10.1016/j.neuropharm.2007.06.030 17714745 PMC2706320

[B10] Chini B. Parenti M. (2004). G-protein coupled receptors in lipid rafts and caveolae: how, when and why do they go there? J. Mol. Endocrinol. 32, 325–338. 10.1677/jme.0.0320325 15072542

[B11] Couet J. Li S. Okamoto T. Ikezu T. Lisanti M. P. (1997). Identification of peptide and protein ligands for the caveolin-scaffolding domain. Implications for the interaction of caveolin with caveolae-associated proteins. J. Biol. Chem. 272, 6525–6533. 10.1074/jbc.272.10.6525 9045678

[B12] D’Antona A. M. Ahn K. H. Kendall D. A. (2006). Mutations of CB1 T210 produce active and inactive receptor forms: correlations with ligand affinity, receptor stability, and cellular localization. Biochemistry 45, 5606–5617. 10.1021/bi060067k 16634642 PMC2667143

[B13] Daigle T. L. Kwok M. L. Mackie K. (2008). Regulation of CB1 cannabinoid receptor internalization by a promiscuous phosphorylation-dependent mechanism. J. Neurochem. 106, 70–82. 10.1111/j.1471-4159.2008.05336.x 18331587 PMC3707135

[B14] Dainese E. Oddi S. Bari M. Maccarone M. (2007). Modulation of the endocannabinoid system by lipid rafts. Curr. Med. Chem. 14, 2072–2715. 10.2174/092986707782023235 17979719

[B15] De Jesús M. L. Sallés J. Meana J. J. Callado L. F. (2006). Characterization of CB1 cannabinoid receptor immunoreactivity in postmortem human brain homogenates. Neuroscience 140, 635–643. 10.1016/j.neuroscience.2006.02.024 16563642

[B16] Dutta D. Williamson C. D. Cole N. B. Donaldson J. G. (2012). PitStop2™ 2 is a potent inhibitor of clathrin-independent endocytosis. PLoS One 7:e45799. 10.1371/journal.pone.0045799 23029248 PMC3448704

[B17] Epand R. F. Thomas A. Brasseur R. Vishwanathan S. A. Hunter E. Epand R. M. (2006). Juxtamembrane protein segments that contribute to recruitment of cholesterol into domains. Biochemistry 45, 6105–6114. 10.1021/bi060245+ 16681383 PMC2515711

[B18] Garcia D. E. Brown S. Hille B. Mackie K. (1998). Protein kinase C disrupts cannabinoid actions by phosphorylation of the CB1 cannabinoid receptor. J. Neurosci. 18, 2834–2841. 10.1523/JNEUROSCI.18-08-02834.1998 9526000 PMC6792582

[B19] Girach F. Craig T. J. Rocca D. L. Henley J. M. (2013). RIM1α SUMOylation is required for fast synaptic vesicle exocytosis. Cell Rep. 5, 1294–1301. 10.1016/j.celrep.2013.10.039 24290762 PMC3898736

[B20] Grimsey N. L. Graham E. S. Dragunow M. Glass M. (2010). Cannabinoid Receptor 1 trafficking and the role of the intracellular pool: implications for therapeutics. Biochem. Pharmacol. 80, 1050–1062. 10.1016/j.bcp.2010.06.007 20599795

[B21] Hsieh C. Brown S. Derleth C. Mackie K. (1999). Internalization and recycling of the CB1 cannabinoid receptor. J. Neurochem. 73, 493–501. 10.1046/j.1471-4159.1999.0730493.x 10428044

[B22] Jin W. Brown S. Roche J. P. Hsieh C. Celver J. P. Kovoor A.. (1999). Distinct domains of the CB1 cannabinoid receptor mediate desensitization and internalization. J. Neurosci. 19, 3773–3780. 10.1523/JNEUROSCI.19-10-03773.1999 10234009 PMC6782730

[B23] Kapur A. Samaniego P. Thakur G. A. Makriyannis A. Abood M. E. (2008). Mapping the structural requirements in the CB1 cannabinoid receptor transmembrane helix II for signal transduction. J. Pharmacol. Exp. Ther. 325, 341–348. 10.1124/jpet.107.133256 18174385 PMC3767288

[B24] Keren O. Sarne Y. (2003). Multiple mechanisms of CB1 cannabinoid receptors regulation. Brain Res. 980, 197–205. 10.1016/s0006-8993(03)02970-6 12867259

[B25] Leterrier C. Lainé J. Darmon M. Boudin H. Rossier J. Lenkei Z. (2006). Constitutive activation drives compartment-selective endocytosis and axonal targeting of type 1 cannabinoid receptors. J. Neurosci. 26, 3141–3153. 10.1523/JNEUROSCI.5437-05.2006 16554465 PMC6674101

[B26] Lutz B. (2009). Endocannabinoid signals in the control of emotion. Curr. Opin. Pharmacol. 9, 46–52. 10.1016/j.coph.2008.12.001 19157983

[B27] Maccarrone M. Guzmán M. Mackie K. Doherty P. Harkany T. (2014). Programming of neural cells by (endo)cannabinoids: from physiological rules to emerging therapies. Nat. Rev. Neurosci. 15, 786–801. 10.1038/nrn3846 25409697 PMC4765324

[B28] Marcu J. Shore D. M. Kapur A. Trznadel M. Makriyannis A. Reggio P. H.. (2013). Novel insights into CB1 cannabinoid receptor signaling: a key interaction identified between the extracellular-3 loop and transmembrane helix 2. J. Pharmacol. Exp. Ther. 345, 189–197. 10.1124/jpet.112.201046 23426954 PMC3629795

[B29] McAllister S. D. Rizvi G. Anavi-Goffer S. Hurst D. P. Barnett-Norris J. Lynch D. L.. (2003). An aromatic microdomain at the cannabinoid CB1 receptor constitutes an agonist/inverse agonist binding region. J. Med. Chem. 46, 5139–5152. 10.1021/jm0302647 14613317

[B30] McDonald N. A. Henstridge C. M. Conolly C. N. Irving A. J. (2007). An Essential role for constitutive endocytosis, but not activity, in the axonal targeting of the CB1 cannabinoid receptor. Mol. Pharmacol. 71, 976–984. 10.1124/mol.106.029348 17182888

[B31] Moreira F. A. Jupp B. Belin D. Dalley J. W. (2015). Endocannabinoids and striatal function: implications for addiction-related behaviours. Behav. Pharmacol. 26, 59–72. 10.1097/FBP.0000000000000109 25369747 PMC5398317

[B32] Nie J. Lewis D. L. (2001). Structural domains of the CB1 cannabinoid receptor that contribute to constitutive activity and G-protein sequestration. J. Neurosci. 21, 8758–8764. 10.1523/JNEUROSCI.21-22-08758.2001 11698587 PMC6762285

[B33] Oddi S. Dainese E. Fezza F. Lanuti M. Barcaroli D. De Laurenzi V.. (2011). Functional characterization of putative cholesterol binding sequence (CRAC) in human type-1 cannabinoid receptor. J. Neurochem. 116, 858–865. 10.1111/j.1471-4159.2010.07041.x 21214565

[B34] Pacher P. Batkai S. Kunos G. (2006). The endocannabinoid system as an emerging target of pharmacotherapy. Pharmacol. Rev. 58, 389–462. 10.1124/pr.58.3.2 16968947 PMC2241751

[B35] Pándy-Szekeres G. Munk C. Tsonkov T. M. Mordalski S. Harpsøe K. Hauser A. S.. (2018). GPCRdb in 2018: adding GPCR structure models and ligands. Nucleic Acids Res. 46, D440–D446. 10.1093/nar/gkx1109 29155946 PMC5753179

[B36] Parton R. G. Simons K. (2007). The multiple faces of caveolae. Nat. Rev. Mol. Cell Biol. 8, 185–194. 10.1038/nrm2122 17318224

[B37] Rimmerman N. Hughes H. V. Bradshaw H. B. Pazos M. X. Mackie K. Prieto A. L.. (2008). Compartmentalization of endocannabinoids into lipid rafts in a dorsal root ganglion cell line. Br. J. Pharmacol. 153, 380–389. 10.1038/sj.bjp.0707561 17965731 PMC2219527

[B38] Rodal S. K. Skretting G. Garred O. Vilhardt F. van Deurs B. Sandvig K. (1999). Extraction of cholesterol with methyl-β-cyclodextrin perturbs formation of clathrin-coated endocytic vesicles. Mol. Biol. Cell 10, 961–974. 10.1091/mbc.10.4.961 10198050 PMC25220

[B39] Rozenfeld R. (2011). Type I cannabinoid receptor trafficking: all roads lead to lysosome. Traffic 12, 12–18. 10.1111/j.1600-0854.2010.01130.x 21040297

[B40] Ruehle S. Rey A. A. Remmers F. Lutz B. (2012). The endocannabinoid system in anxiety, fear memory and habituation. J. Psychopharmacol. 26, 23–29. 10.1177/0269881111408958 21768162 PMC3267552

[B41] Sampo B. Kaech S. Kunz S. Banker G. (2003). Two distinct mechanisms target membrane proteins to the axonal surface. Neuron 37, 611–624. 10.1016/s0896-6273(03)00058-8 12597859

[B42] Sarnataro D. Grimaldi C. Pisanti S. Gazzerro P. Laezza G. Zurzolo C.. (2005). Plasma membrane and lysosomal localization of CB1 cannabinoid receptor are dependent on lipid rafts and regulated by anandamide in human breast cancer cells. FEBS Lett. 579, 6343–6349. 10.1016/j.febslet.2005.10.016 16263116

[B43] Sarnataro D. Pisanti S. Santoro A. Gazzerro P. Malfitano A. M. Laezza C.. (2006). The cannabinoid CB1 receptor antagonist rimonabant (SR141716) inhibits human breast cancer cell proliferation through a lipid raft-mediated mechanism. Mol. Pharmacol. 70, 1298–1306. 10.1124/mol.106.025601 16822929

[B44] Schneider M. Kasanetz F. Lynch D. L. Friemel C. M. Lassalle O. Hurst D. P.. (2015). Enhanced functional activity of the cannabinoid type-1 receptor mediates adolescent behavior. J. Neurosci. 35, 13975–13988. 10.1523/JNEUROSCI.1937-15.2015 26468198 PMC4604232

[B45] Scott C. E. Abrol R. Ahn K. H. Kendell D. A. Goddard W. A. III. (2013). Molecular basis for dramatic changes in cannabinoid CB1 G protein-coupled receptor activation upon single and double point mutations. Protein Sci. 22, 101–113. 10.1002/pro.2192 23184890 PMC3575865

[B46] Shim J. Y. Ahn K. H. Kendall D. A. (2013). Molecular basis of cannabinoid CB1 receptor coupling to the G protein heterotrimer Gαiβγ: identification of key CB1 contacts with the C-terminal helix α5 of Gαi. J. Biol. Chem. 288, 32449–32465. 10.1074/jbc.M113.489153 24092756 PMC3820880

[B47] Shim J. Y. Bertalovitz A. C. Kendall D. A. (2011). Identification of essential cannabinoid-binding domains: structural insights into early dynamic events in receptor activation. J. Biol. Chem. 286, 33422–33435. 10.1074/jbc.M111.261651 21795705 PMC3190901

[B48] Silverman M. A. Peck R. Glover G. He C. Carlin C. Banker G. (2005). Motifs that mediate dendritic targeting in hippocampal neurons: a comparison with basolateral targeting signals. Mol. Cell. Neurosci. 29, 173–180. 10.1016/j.mcn.2005.02.008 15911342

[B49] Silvestri C. Di Marzo V. (2013). The endocannabinoid system in energy homeostasis and the etiopathology of metabolic disorders. Cell Metab. 17, 475–490. 10.1016/j.cmet.2013.03.001 23562074

[B50] Simon A. C. Loverdo C. Gaffuri A. Urbanski M. Ladarre D. Carrel D.. (2013). Activation-dependent plasticity of polarized GPCR distribution on the neuronal surface. J. Mol. Cell Biol. 5, 250–265. 10.1093/jmcb/mjt014 23585691

[B51] Soltesz I. Alger B. E. Kano M. Lee S. H. Lovinger D. M. Ohno-Shosaku T.. (2015). Weeding out bad waves: towards selective cannabinoid circuit control in epilepsy. Nat. Rev. Neurosci. 16, 264–277. 10.1038/nrn3937 25891509 PMC10631555

[B52] Subtil A. Gaidarov I. Kobylarz K. Lampson M. A. Keen J. H. McGraw T. E. (1999). Acute cholesterol depletion inhibits clathrin-coated pit budding. Proc. Natl. Acad. Sci. U S A 96, 6775–6780. 10.1073/pnas.96.12.6775 10359788 PMC21991

[B53] Wager-Miller J. Westenbroek R. Mackie K. (2002). Dimerization of G protein-coupled receptors: CB1 cannabinoid receptors as an example. Chem. Phys. Lipids 121, 83–89. 10.1016/s0009-3084(02)00151-2 12505693

[B54] Wu D. Yang L. Goschke A. Stumm R. Brandenburg L. Liang Y.. (2008). Role of receptor internalization in the agonist-induced desensitization of cannabinoid type 1 receptors. J. Neurochem. 104, 1132–1143. 10.1111/j.1471-4159.2007.05063.x 17986216

